# Evolving robotic surgery training and improving patient safety, with the integration of novel technologies

**DOI:** 10.1007/s00345-020-03467-7

**Published:** 2020-11-06

**Authors:** I-Hsuan Alan Chen, Ahmed Ghazi, Ashwin Sridhar, Danail Stoyanov, Mark Slack, John D. Kelly, Justin W. Collins

**Affiliations:** 1 Division of Surgery and Interventional Science, Research Department of Targeted Intervention, University College London, London, UK; 2 Department of Surgery, Division of Urology, Kaohsiung Veterans General Hospital, No. 386, Dazhong 1st Rd., Zuoying District, Kaohsiung, 81362 Taiwan; 3 Wellcome/ESPRC Centre for Interventional and Surgical Sciences (WEISS), University College London, London, UK; 4 Department of Urology, Simulation Innovation Laboratory, University of Rochester, New York, USA; 5 Division of Uro-Oncology, University College London Hospital, London, UK; 6 University of Cambridge, Cambridge, UK

**Keywords:** Robotic-assisted surgery, Training, Surgical education, 3D printed models, Telementoring, Eye tracking, Patient safety, Proficiency-based progression, Machine learning

## Abstract

**Introduction:**

Robot-assisted surgery is becoming increasingly adopted by multiple surgical specialties. There is evidence of inherent risks of utilising new technologies that are unfamiliar early in the learning curve. The development of standardised and validated training programmes is crucial to deliver safe introduction. In this review, we aim to evaluate the current evidence and opportunities to integrate novel technologies into modern digitalised robotic training curricula.

**Methods:**

A systematic literature review of the current evidence for novel technologies in surgical training was conducted online and relevant publications and information were identified. Evaluation was made on how these technologies could further enable digitalisation of training.

**Results:**

Overall, the quality of available studies was found to be low with current available evidence consisting largely of expert opinion, consensus statements and small qualitative studies. The review identified that there are several novel technologies already being utilised in robotic surgery training. There is also a trend towards standardised validated robotic training curricula. Currently, the majority of the validated curricula do not incorporate novel technologies and training is delivered with more traditional methods that includes centralisation of training services with wet laboratories that have access to cadavers and dedicated training robots.

**Conclusions:**

Improvements to training standards and understanding performance data have good potential to significantly lower complications in patients. Digitalisation automates data collection and brings data together for analysis. Machine learning has potential to develop automated performance feedback for trainees. Digitalised training aims to build on the current gold standards and to further improve the ‘continuum of training’ by integrating PBP training, 3D-printed models, telementoring, telemetry and machine learning.

## Introduction

It is recognised that errors are more common early in the surgeons learning curve [1] and the combination of simultaneously learning about both technology and technique, on patients, has inherent patient safety risks if training is not optimised [2, 3].

The first validated robotic training curriculum was published in 2015 [4]. This validated curriculum is the current gold standard and has been replicated by several societies in multiple specialties [5, 6]. The standardised structure describes staged training commencing with a baseline evaluation, e-learning and operating-room (OR) observation. With modules of simulation training, including wet-laboratory training in cadavers, pigs and other animal models. However, centralised wet-laboratory training centres are expensive and limit access. Another key issue is the level of competence that the trainee has when they commence operations on patients. Expertise may not be available locally, requiring travelling proctors. Weaknesses in individual’s training and subsequent performance can be missed if training is not objectively assessed, benchmarked and quality assured. In the aviation industry, there are international training standards that are benchmarked and quality assured [7]. Proficiency in performance must be shown before the pilot is allowed to fly a plane with passengers onboard. The same rigorous approach to surgical training has not yet been applied [7].

To improve surgical training, we need awareness of weaknesses, quality assured standards and access to affordable training that are integrated with job planning. The combination of systems thinking with a proficiency-based progression (PBP) approach to training has been shown to be highly successful in reducing errors in aviation training [7], whereas surgical training has historically been an apprentice model, with variabilities in the trainer’s skills as both a surgeon and educator [1]. Ultimately, all stages of training will benefit from digitalisation and automated data collection related to surgeon performance.

## Materials and methods

A systematic narrative review was performed with a comprehensive computerised search completed using PubMed and Medline databases. We systematically searched using medical subject headings including ‘robot-assisted surgery training’, ‘robotic surgery training’, ‘curriculum development’ and ‘proficiency-based training’, ‘surgical education’, ‘3D printed models’, ‘telementoring’, ‘eye tracking’, ‘machine learning’ and ‘AI’. Articles of interest included reports of novel technologies used in health-care and surgical training, prospective studies on the impact of robotic simulation training, robotic training curriculum development with validation and systematic reviews on robotic training published between July 2000, when the first robotic systems received FDA approval in the USA [7], and March 2020. Other significant studies cited in the reference list of selected papers were evaluated, as well as studies of interest published before the systematic search. Sections of the review were allocated to six researchers with expertise in that area and the reviewers independently selected papers for detailed review evaluating the abstract and, if necessary, the full-text manuscript. Potential discrepancies for inclusion were resolved by open group discussion.

### Findings

Overall, the quality of available studies was found to be low with current available evidence consisting largely of expert opinion, consensus statements and small qualitative studies. The review identified that there are several novel technologies already being utilised in robotic surgery training and also a trend towards standardised validated robotic training curricula. Currently, the majority of validated curricula follow traditional methods that do not incorporate novel technologies.

### Establishing surgical performance metrics as a starting point

Surgical training has historically been delivered via a master-apprentice model, where the trainee observes and learns from the experienced trainer, eventually being ‘signed off’ as competent. However, subjective assessments of surgical performance have been shown to be highly variable with poor inter-rater reliability [8]. Skills learning is more efficient when sustained deliberate practice (SDP) is enabled [9]. This requires the skills to be defined with objective metrics of performance that are agreed by both the trainer and student [9]. SDP is an important element of PBP training, which has been shown to reduce error rates, early in the learning curve, by approximately 50% [8]. SDP states that repetition of skills with deliberate practice is key to success and that the defined metrics should be able to be replicated in various settings. Objective metrics once established should be utilised in multiple training settings to enable a continuum of training, from e-learning that describes the metrics in key index procedures, to the development of the simulation models that reflect the metrics [10], to telementoring protocols, to recording and auditing outcome data. There is good evidence that when surgery is standardised, it is easier to identify the subtleties of the technique to improve patient outcomes [11], and to identify postoperative complications related to sub-optimised technique [12].

**Fig. 1:**
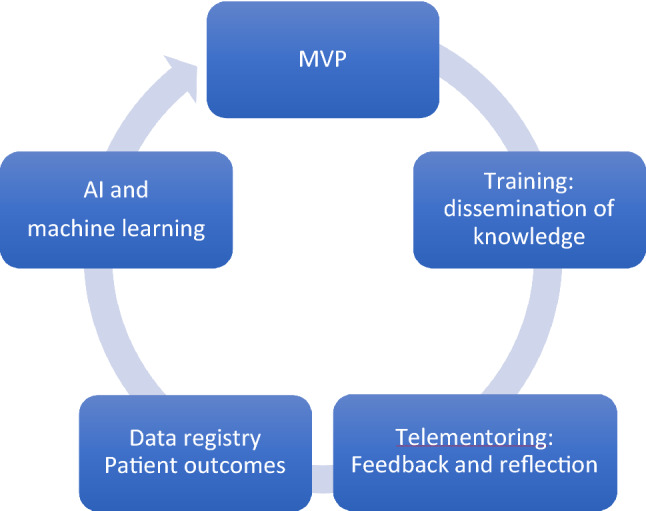
Defining standardised objective performance metrics is an ideal starting point

Various approaches to defining metrics have been defined. The process requires task deconstruction and identification of key elements. Essential elements within each defined phase of the procedure include the tasks to be completed and the errors to avoid. To enable SDP, it is important to have agreement between the trainer and trainee. Consensus on the phases, tasks and errors can be reached using the Delphi process [13]. Strategies to drive standardised training, with a top-down approach, include ‘train-the-trainer’ courses, where trainers learn about the curriculum structure and content, the metrics to assess and how to deliver training safely [1]. An evolving surgical technique is equally likely to be affected by technological advancements in surgery and both optimisation of technique and technology benefit from standardisation of the fundamentals. This will be increasingly utilised with the development of robotic networks and integration of artificial intelligence (AI) and machine learning [14], to scale deep learning development into a more predictable and efficient practice benefits from standardisation. It is a common language or a coordination mechanism for industry, academics and clinicians to accelerate the development of both the technology and technique. Thus, a standardised approach to a key index procedure could be described as a minimal viable product (MVP), rather than a defined end product of established optimised performance (See Fig. 1).

### E-learning

**Fig. 2:**
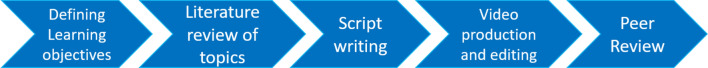
Development of educational video content

Online education has potential to deliver both synchronous and asynchronous learning. Video content is ‘richer’ than text and previous research concludes 1 min of video is worth 1.8 million words. The reasoning is: “if ‘a picture is worth a thousand words’, then a minute of video with 30 frames per second is worth at least 1.8 million words.” [15]. Cost models estimate the costs of developing video on demand for education according to Levin’s Ingredient, model related to personnel, equipment and consumables [16]. Video content development can be planned according to Fig. 2.

Future developments in innovative educational content can be broadly considered as platform functionality related and approaches to enhance engagement, although there is significant overlap. Virtual reality (VR) simulation is already widely available and has been shown to improve patient outcomes, but is not yet fully integrated into training [17]. Although the research concerning the implementation of augmented reality (AR) in surgical education is relatively limited, there are promising results regarding the teaching potential [18]. There is also evidence that gamification of knowledge acquisition is beneficial to learning curves and this is currently being explored in surgical training [19]. Further research is needed to prove if AR and VR can effectively replace or supplement traditional surgical pedagogy methods and whether gamification promotes a desire to learn.

### Telepresence

In the current robotic training, without telementoring, an experienced surgeon usually proctors the trainee in their own hospital for their first five to ten operations [4]. If the hospital commencing robotic surgery lacks expertise, then this requires surgeons from centres of excellence to travel to that hospital. Proctorship is therefore expensive and supervision limited to an agreed number of operations. With the likely possibility of the ‘inexperienced’ surgeon being confronted with unusual anatomy or a difficult case after this period of proctorship, this is the time that the surgeon and his/her patient are most vulnerable to the early learning curve. The introduction of telementoring, where training and expert support/guidance can be continued remotely from centres of excellence, is more cost-efficient and accessible [14]. Telementorship consists of audio-visual communication where the remote surgeon can see the operating field, and additional functionalities to aid communication include telestration and image overlay [20].

Several studies have concluded telementoring is an effective training tool [21]. One study showed that residents in the telementoring group performed significantly better compared to non-mentoring group (*p* < 0.001) [22]. The safety of telementoring has also been established. In a systematic review of 11 studies, 9 concluded that telementoring did not prolong surgery time compared to on-site mentoring; none of them reported an elevated complication rate; and only 3% of the total number of cases reported technical issues [23]. Of note, a study of laparoscopic cholecystectomies conducted by Byrne et al. [24] concluded that telementoring could be used as bridge between on-site supervision to totally unsupervised performance. A telementoring study by Pahlsson et al. [25] focused on endoscopic retrograde cholangiopancreatography (ERCP) confirmed that telementoring might be delivered from a high-volume endoscopist at a tertiary hospital to a low-volume rural hospital, to provide a higher success rate which could be maintained without telementoring support.

One of the original purposes of telementoring was for the battlefield and potentially the biggest advantages may be in the management of emergency scenarios [26]. In 1999, Cubano and colleagues successfully connected USS Abraham Lincoln aircraft with a land-based surgical mentor and completed five laparoscopic hernia repairs under telementoring guidance [27]. Telementoring can currently be delivered with different network infrastructure, including wide access networks (WANs) and 5G [28]. Project 6 was proposed by the Society of American Gastrointestinal and Endoscopic Surgeons (SAGES) in 2015, aiming to promote development of surgical telementoring [29]. A current limitation is surgical telementoring’s requirement for bandwidth and rural areas usually lack it. With the development of 5G, this barrier would likely be overcome [28].

### Eye tracking

In general, the mental workload associated with an easy task is low, whereas difficult tasks produce higher mental workload. Mental workload can also be described by the difference between task demands and available attention resources. Thus, a high workload task that is mentally demanding leaves little or no spare attention capacity to deal with new or unexpected events, and the less likelihood of learning. Increased workload during surgery has been associated with inferior task performance, a higher likelihood of errors and the possibility of an incomplete skill transfer to the clinical environment [30, 31]. Until recently, cognitive (mental) load measures were limited to subjective ratings administrated after the task (NASA-TLX), or performance on a secondary-task as opposed to instantaneous load in a given moment [32]. While such measures are well suited to evaluating the relative differences in cognitive load between practice trials, they cannot provide detailed information about whether increased or decreased load is experienced during the performance of a learning task. Task-evoked pupillary responses (TEPRs) that include changes in pupil diameter and patterns in eye movement fixation have been found to occur shortly after the onset of a task and subside quickly after processing is terminated. Studying changes in TEPRs traditionally requires complex and limiting laboratory infrastructure with non-mobile cameras and onerous manual data collection and analysis.

Newly developed portable devices have facilitated this process, as they allow digital recording of pupil changes and a more convenient means for the quantification of TEPRs in dynamic environments. These devices have demonstrated that physicians with more training and experience exhibit less cognitive load than novices when answering questions in their field of expertise [33]. Recently, a publication evaluated the relationship between eye-tracking measures and perceived workload in robotic surgical tasks. Eight surgical trainees performed up to 12 simulated exercises. Pupil diameter and gaze entropy were found to distinguish differences in workload between task difficulty levels, and both metrics increased as task level difficulty increased. It was found that eye-tracking features achieved an accuracy of 84.7% in predicting workload levels [34]. Causer et al. [35] also maintained that gaze training could help trainees lessen the negative effects of anxiety by concentrating on continually relevant information. In future research, we can utilise a lightweight, non-obtrusive eye tracker (Pupil Labs) worn by the participants to evaluate the impact of various robotic training curricula on the trainee’s workload. Owing to the fundamental differences of gaze behaviour between experts and novices, eye-tracking technology also has potential for proficiency assessment [34].

**Fig. 3:**
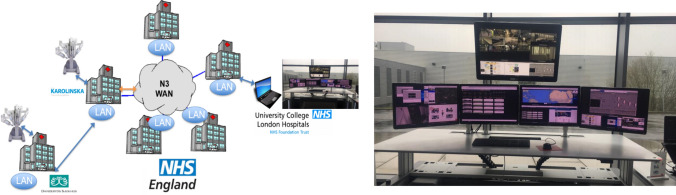
A telementoring hub could potentially be linked to multiple hospitals, both nationally and internationally

Eye tracking has been shown to be a valuable training tool that can impact the learning curve. Chestwood et al. demonstrated that the projection of an expert’s gaze pattern on a trainee’s laparoscopic screen during a simulation task was found to aid trainee performance. The research concluded that, by simultaneously reflecting a supervisor’s gaze to a trainee, the completion time of laparoscopic tasks and number of errors could be significantly reduced [36]. Additionally, eye tracking can measure the gaze focusing on an area of interest (AOI). One study indicated that as medical students became more familiar with anatomical landmarks, cognitively salient gaze pattern changes within AOI could be observed and so progression of gaze behaviour may be expected during active learning and familiarisation [37]. In future work assessing telementoring, we could assess the use of the projected eye tracking of a trainer to aid in remote proctoring of trainees. Eye tracking can be utilised with both an open console and immersive console (Fig. 3).

### Fabricating the ideal surgical simulation platform utilising 3D printing

One area of simulation that has proven to be difficult is the creation of a high-fidelity process that accurately and reproducibly simulates an entire procedure including relevant anatomy and pathology (see Table 1). With increased awareness of the benefits of standardised training, a novel full-immersion simulation using 3D printed models builds on the principles of SDP, with integration of defined performance metrics [10].

**Table 1:** Comparison of the different surgical training models

Model	Strengths	Weaknesses
Task deconstruction models	Address metrics and are cost effective, e.g. chicken gizzard model for vesico-urethral anastmosis	Limited development to comprehensively address metrics, benchmarks and error management
Porcine model	Flexible training model for tissue handling	Expensive
		Not human anatomy
		No human pathology
		Limited accessibility
Canine cadaver model	Flexible training model for tissue handling	Not human anatomy
		No human pathology
		Limited accessibility
Human cadaver model	Flexible training model	Expensive
		Lacks pathology and does not bleed
		Limited accessibility
3D printed models [10]	Flexible training model	Currently, high development costs (lowered if printed casts rather than printed models)
	Can incorporate pathology and vascularisation	
	Increasingly realistic tissue handling	Models that address specific defined metrics need to be developed
	Can incorporate metrics and benchmarks [10]	
VR simulation	Advanced procedural training models available (e.g. robotic prostatectomy, hysterectomy)	Current scope/range/image quality limited
AR simulation	Potential to develop	Limited development

**Fig. 4:**
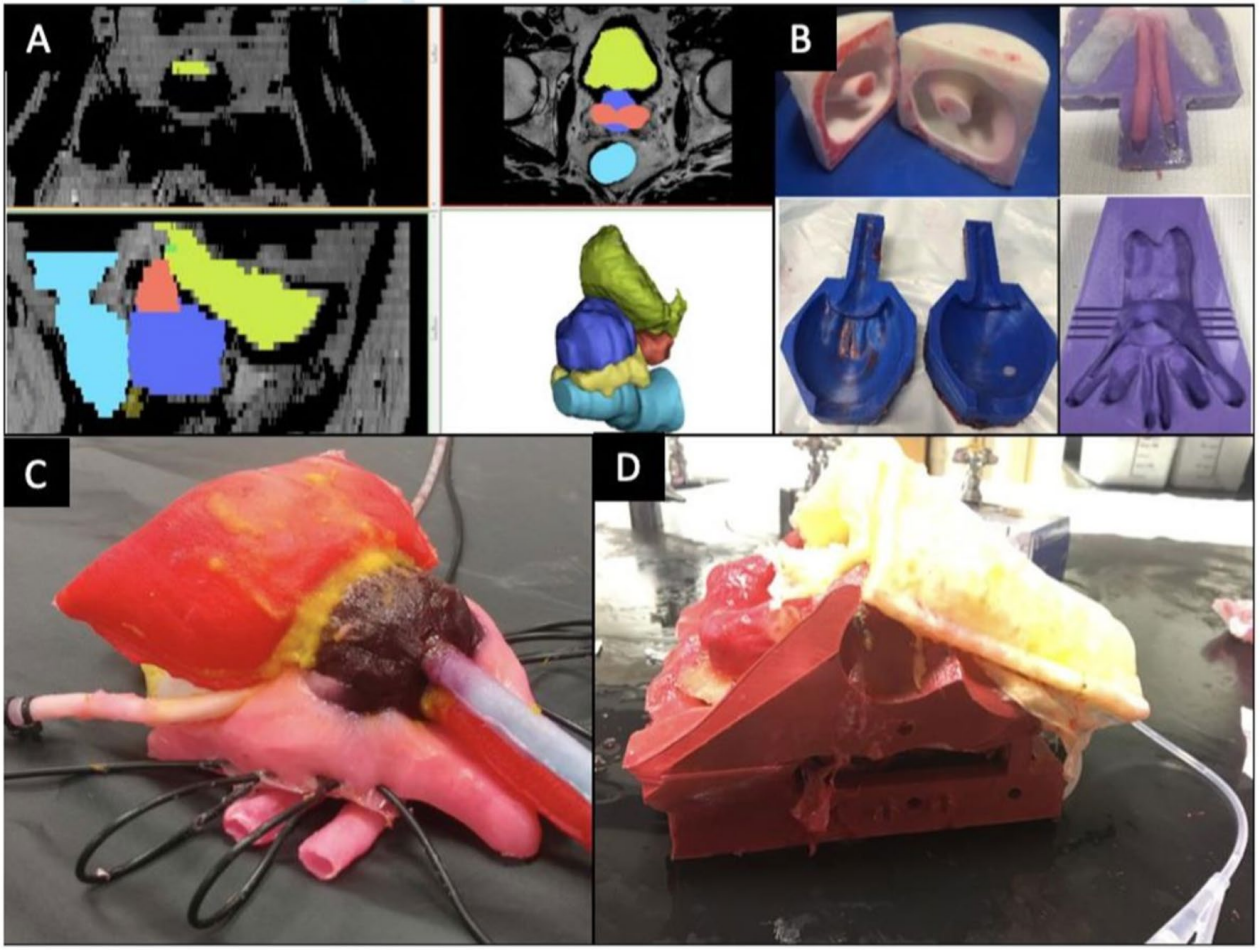
Construction of nerve-sparing robot-assisted radical prostatectomy (NS-RARP) simulation platform

The Simulation Innovation Laboratory at the University of Rochester Medical Centre has successfully merged polymer casting and 3D printing technologies to fabricate anatomically accurate training platforms permitting realistic dissection, haemostasis and suturing for complex procedures as percutaneous nephrolithotomy [38], robot-assisted radical prostatectomy (RARP) [10] and partial nephrectomy [39]. Taking the RARP model [10] as an example, the MRI of a patient with T1c, Gleason 7 cancer was imported into Mimics 20.0 (Mimics; Materialise, Leuven, Belgium)., for segmentation of each of the patient’s pelvic organs to form a computer-aided design (CAD) anatomical model (Fig. 4a). Individual injection casts were then designed from the CAD and 3D printed using a Fusion3 F400-S 3D printer (Fusion3 Design, Greensboro, NC, USA) (Fig. 4b). PVA hydrogel was injected into these casts based on the desired mechanical properties for each organ and cast in series to replicate their anatomical relationships between various organs (Fig. 4c). The model was perfused through hollow, watertight vessels incorporated into the NVB, dorsal venous complex and prostatic pedicle during the casting process. To replicate the entire procedure, the prostatectomy organ complex was layered in its anatomical configuration within a 3D-printed male pelvis that was fitted with pelvic floor muscles, pelvic fat, and relevant structures made of PVA (Fig. 4d).

**Fig. 5:**
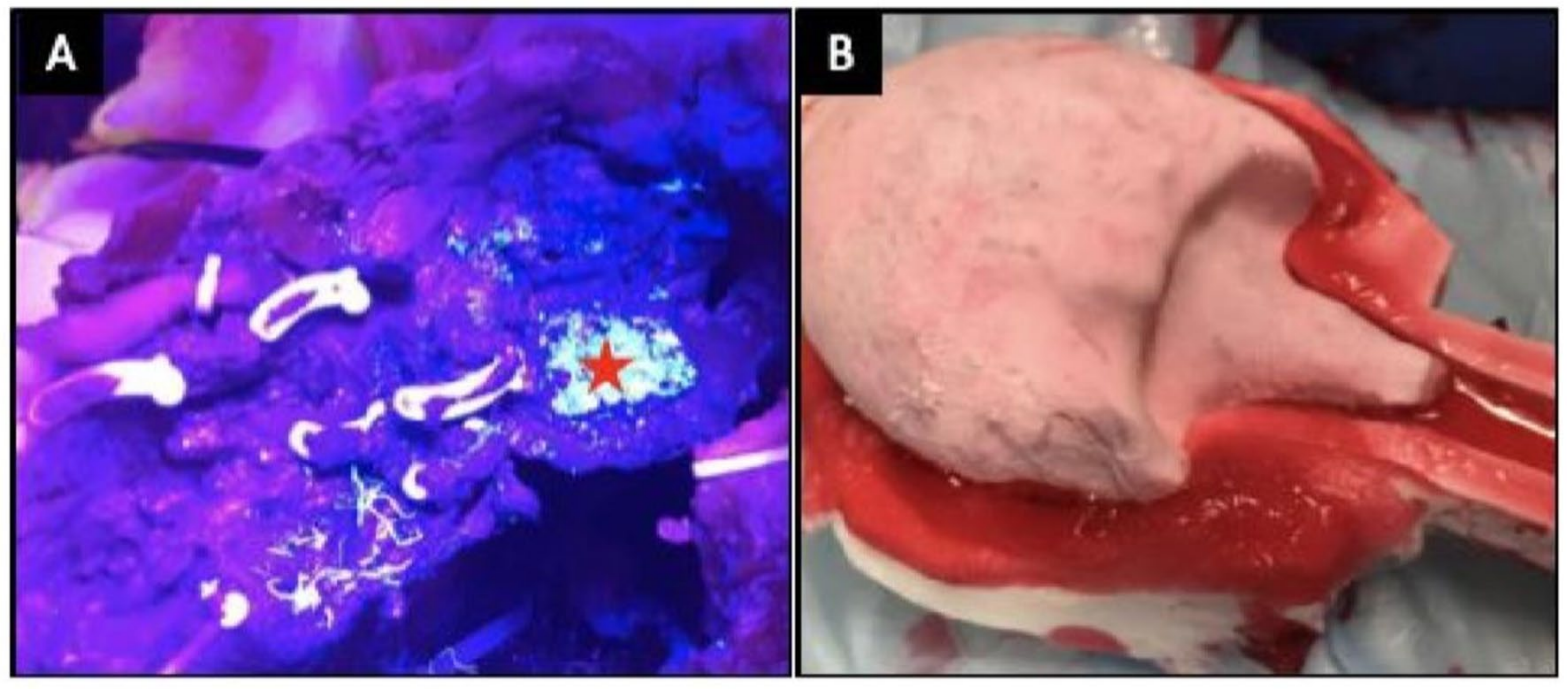
Demonstration of positive margin (**a**) and bladder neck (**b**) in NS-RARP simulation platform

Training and assessment go hand in hand, training without formative feedback is reduced to sheer repetition [9]. Naturally, the best method of assessment would be one of clinical relevance that could be tracked to evaluate progress during training. Our casting technique allowed incorporation of Clinically-Relevant Metrics Performance Metrics of Simulation (CRPMS) pertinent to an RARP procedure including key phases of the procedure: nerve tension during NVB dissection (measured through calibrated analogue stretch sensors, aligned within the NVB during the casting process); surgical margins (measured by addition of a chemical that exhibits chemiluminescence in the prostate cast); and vesico-urethral anastomosis (VUA) integrity (that could be tested for any leaks after injecting 180 cc of saline) (Fig. 5).

Simulations by five experts and nine novices were completed to validate incorporated CRPMS and correlate them to standardised objective assessments, e.g. global evaluative assessment of robotic skills (GEARS) and robotic anastomosis competency evaluation (RACE) [10]. Nerve forces applied during the simulation were significantly lower for experts. Higher force sensitivity (Subcategory of GEARS Score) and Total GEARS Score correlated with lower nerve forces applied with total energy (J) – 0.66 (0.019) and − 0.87 (0.000), respectively, which was significantly different between novices and experts (*p* = 0.003). The VUA leak rate highly correlated with total RACE score − 0.86 (0.000), which was also significantly different between novices and experts (*p* = 0.003). This study presents a novel method for real-time assessment and feedback during robotic surgery training utilising incorporated CRPMS. These efforts provided a platform for reproducing realistic procedural models with the added capacity to provide objective procedural metrics that permit real-time feedback and assessment during robotic surgery training.

### Automated performance metrics

Robotic surgery training curricula aim to objectively assess surgical trainee basic robotic surgical skills or specific procedures using tools such as global skills assessment, robotic-objective structured assessment of technical skills (R-OSATS), global operative assessment of laparoscopic skills (GOALS) and GEARS. While the majority of these tools were utilised for evaluation of basic robotic surgery performance, the GEARS assessed selective steps of specific procedures and associated them with patient outcomes [40]. Nevertheless, these tools counted on human rating, inevitably mingled with subjective bias. In addition, the time and resources of educators or assessors is required. As such, automated performance metrics (APMs) have potential to objectively and analytically evaluate surgical techniques, providing surgical trainees with more equitable and quantifiable assessment across disciplines. Heterogeneity exists in automated methods of surgical skill assessment. Levin et al. [41] identified a pathway of automated methods, including three sessions: data extraction (kinetic and computer vision methods), automated methods (motion tracking of tools, hands and eyes, and muscle contraction analysis), and analysis utilising machine learning, deep learning or performance classification. The procedural approaches most frequently studied were robotic and laparoscopic minimally invasive surgery. The most common utilised method for automation was tool motion tracking. Most previous studies were accomplished in a simulated environment, instead of in the OR.

In the laboratory setting or live surgery, APMs can be categorised into three sectors, encompassing kinematic data (e.g. instrument moving velocity, travelling distance, deceleration and acceleration), systems event data (e.g. master clutch use, camera movement, energy application and third arm swap), and instrument grip force [42]. There are a variety of recording devices, such as ProMISTM, trakSTAR, AcceleGlove, and dVLogger (Intuitive). The most frequently investigated and validated APMs are kinematic data. Both kinematic and systems events data have shown good discrimination between surgeon’s expertise. Although instrument grip force was less frequently studied, Gomez et al. [43] identified that novice robotic surgeons applied higher grip forces than experts when performing laboratory exercises. If these surgical activity recognition models could assess each phase of a surgical procedure, they could be utilised to compute efficiency metrics. Nevertheless, most models reported in the literature ranged from around 50–80% accuracy [44]. Recognition of anatomical landmarks is a current limitation of APMs evaluating surgical skills. Nosrati et al. [45] proposed a new technique to localise both visible and occluded anatomical structures on an endoscopic view of partial nephrectomy. They leveraged both preoperative 3D computed tomography scans and intraoperative endoscopic visual clues as well as vasculature pulsation in order for accurately segmenting the highly fluctuated environment in minimally invasive surgeries.

### AI and machine learning (ML)

Quantifying and evaluating surgical ability are intensely researched topics in the medical community and particularly in minimally invasive surgery. AI driven systems are emerging mostly based on vision (endoscopic video), which embeds space–time information about both the instrument motion and the surgical site that can provide richer discriminative power. Because video is inherently available in many modern surgical procedures and captures a rich log of events, it is a promising digital record that can be analysed to infer ability, but of course it can also be complemented with additional information from integrated ORs or from robot kinematics [46]. Crucial to exploiting video or other OR data for assessing competence is the automatic analysis of the signal, because it is impractical to scale systems relying on manual observations of vast volumes of procedures. For automation, various computer vision (CV) or ML algorithms have been introduced to evaluate surgical performance and also to extract higher level information from the surgical video stream [47]. Both model-based [48, 49] and data-driven [39] algorithms have been explored as a means to lift skill metrics from video akin to other AI application areas, and data-driven and especially deep learning-based methods have rapidly emerged as the most effective and robustly performing algorithms for understanding surgical video.

To extract APMs from video, a number of building block algorithmic capabilities are important to develop, for example: detection of surgical instrument presence [50], delineation of surgical tools’ position and motion [51], segmentation of surgical site into objects [52] or the video into key surgical steps [53, 54], activity or significant event detection [55] as well as others like the detection of critical structures [56]. Marked progress in each of these building blocks of surgical process understanding has taken place in recent years, but a significant challenge is still the availability of large, well annotated datasets that can be used to evaluate systems in a fair and comparable manner. This is particularly lacking for data that combines OR information despite recent efforts or on robotic information and video to support fused analysis [57] with the only currently available data being on phantom environments [58]. The transferability of systems across different operating techniques, instrument toolsets or even different procedures is also largely unmet at present.

With maturity of AI systems a number of other capabilities have also been explored like the estimation of the remaining procedural time form the real-time video feed [59] or automatic image to video retrieval [60] or potentially risk estimation [61]. An important area of work that has received limited attention at present is how such AI powered technologies for understanding surgical process and performance can be utilised in situ with effective user interfaces that appropriately support clinical workflow. They reported that the RF-50 algorithm provided the best performance, delivering 87.2% accuracy in predicting length of stay following RARP. However, there was lack of task-based, efficiency metrics. They demonstrated a better performance for surgical activity recognition in RARP, proving the feasibility of automated postoperative efficiency reports, especially for critical tasks in a clinical procedure. A deep-learning network, an artificial neural network with significant layers, has assisted in discovering hidden and abstract implications present in data. Despite great performance of deep-learning methods in multiple fields, the lack of solid theory explaining how they work would be an important concern. Baghdadi et al. [62] also described ML-based analysis of textural and colour visual features on a robotic endoscopic view to localise anatomical landmarks during RARP. Nevertheless, it is still laborious and time-consuming to label all anatomical landmarks in a surgical procedure to feed data into ML algorithms. How to enable precise automatic annotation by AI rather than repetitive manual work remains a fundamental issue.

## Discussion

It has previously been estimated that 10–15% of surgical patients in the UK have adverse events whilst in hospital. 50% are in the operating room and 50% are preventable [63]. A recent study from the USA estimated that a third of all medical injuries are due to error [64]. The risk of errors has potential to increase with new technologies. In the US between 2000 and 2013 10,624 adverse events related to robotic procedures were reported [2]. During this period, 144 deaths (1.4% of the 10,624 reports), 1391 patient injuries (13.1%), and 8061 device malfunctions (75.9%) were reported. The USA ‘Agency for healthcare research and quality’, estimated that the annual cost to US healthcare from medical errors is €17.1 billion. Six of the top ten identified medical errors are related to surgical procedures [6]. Rigorous analysis of surgical errors and training to reduce their occurrence will reduce the financial burden to both patients and hospitals. However, the majority of surgical training remains opportunistic, unstructured and delivered in an apprenticeship style.

In 2013, a group of experts expressed concern that robotic surgery training is not standardised and insufficient to ensure patient safety [3]. Two years later, the ECRI institute published their annual independent review on health technology hazards, in which a lack of robotic surgical training was identified as one of the top ten risks to US patients [65]. The ECRI report stated: “Insufficient training of surgeons on robotic technologies can result in surgical errors that lead to prolonged surgery, substandard operation outcomes early in the surgeons’ learning curve, complications that require additional treatment and even serious patient injury or death. Errors can result if training is insufficient or ineffective e.g. if it does not provide an assurance of competency”.

Simulation training, which aims to avoid patients being exposed to the trainees’ early learning curve, could be significantly improved by utilising 3D printed models that bleed and incorporate metrics of surgical performance to aid SDP, real-time assessment and feedback [10]. These models also do not require wet-laboratory facilities, thereby reducing costs and making training more accessible. Novel technologies have potential to support SDP in various settings. An evolved digitalised curriculum could be monitored from a centralised hub and incorporate: standardised asynchronous and synchronous e-learning modules with PBP training [8] that has benchmarks, 3D-printed models [10], telemetry [66], eye-tracking metrics [34] and video performance analysis completed with telementoring in real time [29]. All training aspects aligned with defined metrics and delivering a continuum of training. Incorporating supervised ML algorithms to identify and prioritise key elements of training and performance will enable personalised learning and eventually automated performance feedback [14, 66].

## Conclusions

Improvements to training standards and understanding performance data have huge potential to significantly lower complications in patients. Digitalisation automates data collection and brings data together for analysis. Digitalised training aims to build on the current gold standards and to further improve the ‘continuum of training’ by integrating PBP training, 3D-printed models, telementoring, telemetry and machine learning. Study and evaluation of performance metrics and patient outcomes with machine learning have potential to develop automated performance feedback for trainees.

Objective performance metrics will help deliver society-approved and validated robotic surgery curriculums for multiple surgical specialities. This will aid credentialing of surgeons in new medical technologies and their applications.

## Data Availability

This review was completed in sections according to the expertise of the individuals in the group. We have accumulated all papers which were screened, excluded and assessed by these various experts, according to the PRISMA guideline. Current evidence for training technologies includes telementoring, 3D printed models, telemetry, eye tracking, AI and machine learning.
